# Transcriptome based identification of mouse cumulus cell markers that predict the developmental competence of their enclosed antral oocytes

**DOI:** 10.1186/1471-2164-14-380

**Published:** 2013-06-07

**Authors:** Giulia Vigone, Valeria Merico, Alessandro Prigione, Francesca Mulas, Lucia Sacchi, Matteo Gabetta, Riccardo Bellazzi, Carlo Alberto Redi, Giuliano Mazzini, James Adjaye, Silvia Garagna, Maurizio Zuccotti

**Affiliations:** 1 Laboratorio di Biologia dello Sviluppo, Dipartimento di Biologia e Biotecnologie ‘Lazzaro Spallanzani’, Universita’ degli Studi di Pavia, Pavia, Italy; 2 Department of Vertebrate Genomics. Max-Planck Institute for Molecular Genetics, Molecular Embryology and Aging Group, Berlin, Germany; 3 Centro di Ingegneria Tissutale, Universita’ degli Studi di Pavia, Pavia, Italy; 4 Dipartimento di Ingegneria Industriale e dell’Informazione, Universita’ degli Studi di Pavia, Pavia, Italy; 5 Istituto di Genetica Molecolare – Consiglio Nazionale delle Ricerche, Via Ferrata 9, Pavia, Italy; 6 Institute for Stem Cell Research and Regenerative Medicine, Medical Faculty, Heinrich Heine University, Düsseldorf, Germany; 7 Centro di Eccellenza in Biologia Applicata, Universita’ degli Studi di Pavia, Pavia, Italy; 8 Dipartimento di Scienze Biomediche, Biotecnologiche e Traslazionali (S.Bi.Bi.T.), Sezione di Anatomia, Istologia ed Embriologia, Universita’ degli Studi di Parma, Via Volturno, Parma, 39-43125, Italy

**Keywords:** Oocyte, Developmental competence, Cumulus cells, Transcriptome

## Abstract

**Background:**

The cumulus cells (CCs) enveloping antral and ovulated oocytes have been regarded as putative source of non-invasive markers of the oocyte developmental competence. A number of studies have indeed observed a correlation between CCs gene expression, embryo quality, and final pregnancy outcome. Here, we isolated CCs from antral mouse oocytes of known developmental incompetence (NSN-CCs) or competence (SN-CCs) and compared their transcriptomes with the aim of identifying distinct marker transcripts.

**Results:**

Global gene expression analysis highlighted that both types of CCs share similar transcriptomes, with the exception of 422 genes, 97.6% of which were down-regulated in NSN-CCs *vs.* SN-CCs. This transcriptional down-regulation in NSN-CCs was confirmed by *q*RT-PCR analysis of CC-related genes (*Has2, Ptx3, Tnfaip6* and *Ptgs2*). Only ten of the 422 genes were up-regulated with *Amh* being the most up-regulated in NSN-CCs, with an average 4-fold higher expression when analysed by *q*RT-PCR.

**Conclusions:**

The developmental incompetence (NSN) or competence (SN) of antral oocytes can be predicted using transcript markers expressed by their surrounding CCs (i.e., *Has2, Ptx3, Tnfaip6, Ptgs2* and *Amh*). Overall, the regulated nature of the group of genes brought out by whole transcriptome analysis constitutes the molecular signature of CCs associated either with developmentally incompetent or competent oocytes and may represent a valuable resource for developing new molecular tools for the assessment of oocyte quality and to further investigate the complex bi-directional interaction occurring between CCs and oocyte.

## Background

Based on their chromatin morphology, fully-grown antral oocytes may be classified into two distinct types: surrounded nucleolus (SN) oocytes, that present a ring of Hoechst-positive chromatin around the nucleolus and non-surrounded nucleolus (NSN) oocytes, that lack this ring and have a more widespread chromatin [1-6]. These two types of antral oocytes have been described in most of the mammals studied, including mouse [1-3], cow [7], monkeys [8], pigs [9], rats [10] and humans [11,12]. In the mouse and bovine, the two chromatin organizations reflect distinct developmental competence. When matured *in vitro* to metaphase II (MII) and fertilised, SN oocytes may reach the blastocyst stage [4,7] and complete development to the birth of new individuals [13], whereas NSN oocytes cease development at the 2-cell stage [4,13,14].

This protocol of antral oocyte classification, though allowing the recognition of gametes that are potentially developmentally competent or incompetent, has several limitations. First, to allow fluorochrome loading and microscope observation, it requires that oocytes are isolated from its companion cumulus cells (CCs), hence hampering the quality of the following culture steps to the MII stage. Furthermore, although the use of the supravital fluorochrome Hoechst 33342 and the subsequent steps of classification under UV light do not alter significantly the oocytes developmental competence [4], for obvious reasons the employment of this DNA-binding molecule is a strong limitation and is not advisable, outside the laboratory routine with model animals, for the selection of oocytes of domestic species or of humans.

The use of non-invasive markers that permit the classification of SN and NSN oocytes would clearly be more appropriate. To this regard, the cumulus cells (CCs) that envelope fully-grown antral and ovulated oocytes have been considered as putative source of non-invasive molecular markers of the oocyte developmental competence and a number of studies have revealed a correlation between CCs gene expression and embryo quality (for a review see [6]). In all these studies, the oocyte developmental competence was inferred *a posteriori*, by observing the quality of preimplantation embryos obtained and/or the final pregnancy outcome.

The objective of this investigation is the identification of marker transcripts that would allow the non-invasive selection of NSN and SN oocytes. To this regard, we isolated CCs from fully-grown antral mouse oocytes of known developmental incompetence (NSN-CCs) or competence (SN-CCs) and compared by microarrays their whole transcriptome and, by quantitative Real-Time PCR (*q*RT-PCR), the expression profile of CC-related genes (*Has2, Ptx3, Tnfaip6* and *Ptgs2*). We described a group of regulated genes that represent the molecular signature of CCs associated either with developmentally incompetent (NSN) or competent (SN) antral oocytes.

## Results and discussion

### The microarray analysis identified a group of differentially expressed genes as putative markers of the oocyte developmental competence

A strong point of the model study adopted here is that we knew in advance the developmental capability of the antral oocytes from which we isolated the CCs, thus allowing, for the first time, a direct correlation of the transcriptome of the CCs with the oocyte quality.

Out of 25697 probes (from now on named genes) analysed with an Illumina microarray chip, after setting a fold-change cut-off of > 2.0 and a *p*-value of < 0.01, 47 genes were differentially expressed in the comparison between NSN-CCs *vs*. SN-CCs; of these, 46 were down-regulated and only one (*Amh*) was up-regulated (Additional file 1, spreadsheet 1). Then, to verify whether this down-regulated transcriptional profile was a feature of the NSN-CCs, we lowered the fold-change cut-off to > 1.3, maintaining a *p*-value of < 0.01. A total of 422 genes were found differentially expressed in the comparison between NSN-CCs vs. SN-CCs (Additional file 1, spreadsheet 2). None of these genes were expressed exclusively in one of the two types of CCs and, again, the great majority (412, 97.6%) were down-regulated in NSN-CCs, except for 10 genes that were up-regulated, with *Amh* still being the most highly up-regulated gene.

**Figure 1:**
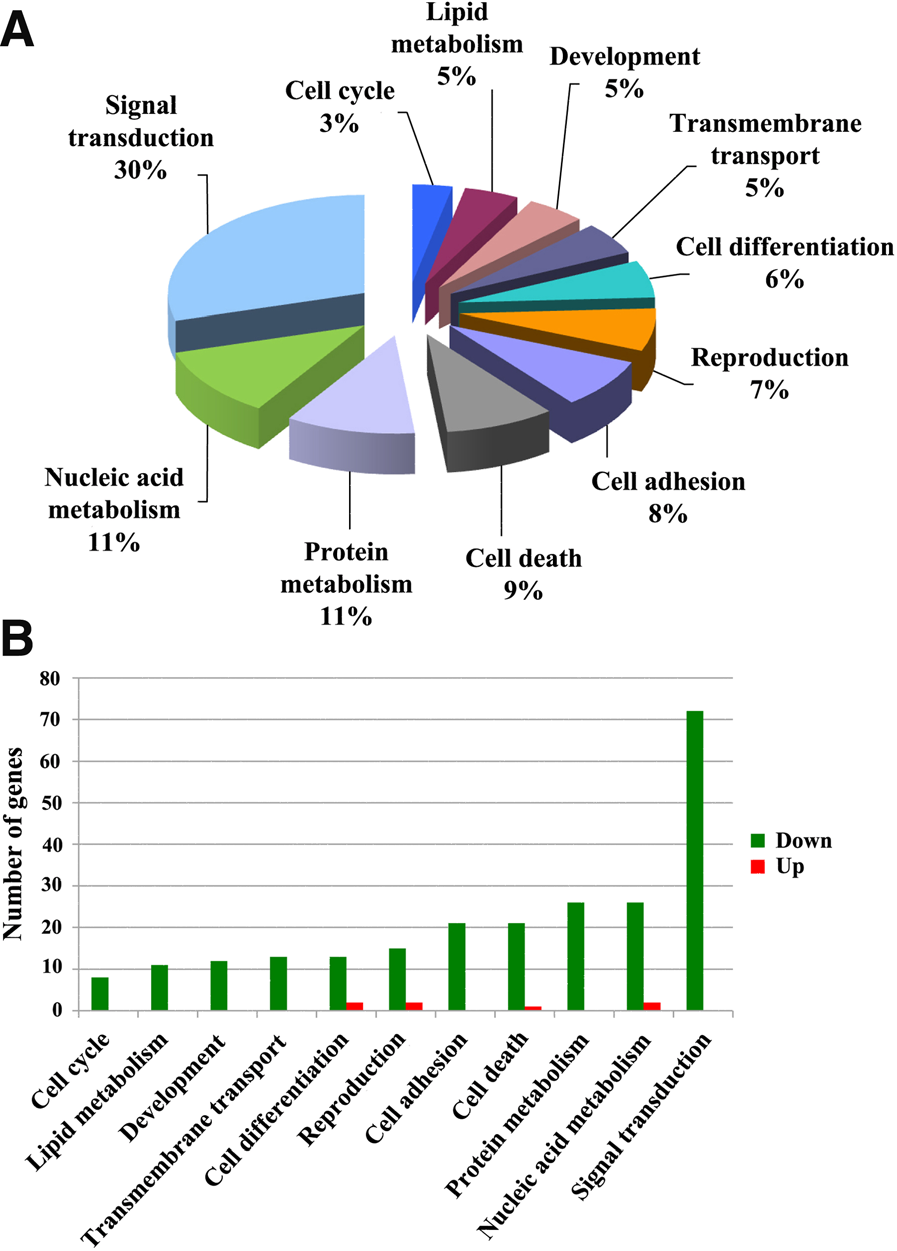
**Microarray-based analysis of the transcription profile of cumulus cells (CCs) isolated from developmentally incompetent (NSN-CCs) or competent (SN-CCs) oocytes.** (**A**) Major biological processes and functions found when comparing the transcription profile of NSN-CCs *vs.* SN-CCs. (**B**) Number of up- and down-regulated genes in each of these processes.

Next, on the differentially expressed genes we performed both a bioinformatic analysis and a bibliographic search of genes known for their association with the ovarian follicle function. A Gene Ontology (GO) enrichment analysis, using the tool provided by the data mining and bioinformatics software Orange (http://orange.biolab.si/), allowed the identification of 11 major biological processes (Figure 1) comprising 247 of the differentially expressed genes (Table 1), the remaining 175 being sequences whose function is yet unidentified. Thirty per cent of these genes, all down-regulated, were assigned to the ‘Signal transduction’ biological process; the only 7 up-regulated genes were attributed to ‘Nucleic acid metabolism’ (*Giyd2* and *Hspa1a*), ‘Cell death’ (*Lgmn*), ‘Cell differentiation’ (*Erdr1* and *Vim*) and ‘Reproduction’ (*Cited2* and *Amh*).

Then, to determine whether and how individual genes are interrelated or interact with each other and to search for biological pathways and the inter-relationships between network genes, with Orange we interrogated the MeSH and STRING repositories. This analysis generated a single broad network made of 142 genes (Additional file 2; Additional file 3) in which the gene *Pttg1* (Pituitary

 tumor-transforming gene 1) stood out with the highest number of neighbours, as shown in Additional file 2, where *Pttg1* is related to its two more proximal neighbours. Interestingly, *Pttg1* emerged as a regulated gene also in a previous microarray study of cow follicles. As in our NSN-CCs, *Pttg1* was down-regulated in CCs isolated from immature *vs.* mature cow follicles [15], indicating that its down-regulation may be correlated with a lower degree of maturity of the follicle enclosing NSN oocytes [3]. The significance and role of this gene network within the biology of the CCs is, at this stage, unknown, although its identification is a prerequisite towards more functional studies that will improve our limited knowledge of both genes and associated pathways in force in these cells.

Finally, we searched the public database PubMed for links between a number of MeSH terms correlated with the ovarian function (Additional file 4) and the 422 differentially expressed genes. Of these, 119 could not be found in the interrogated database, whereas the remaining majority of 303 scored at least one result and 26 obtained the highest score (Additional file 5), with *Amh* and *Has2* at the top of the list. These results highlight the follicle-related nature of this gene list and its possible future use to further our understanding of the interactions between the female gamete and its associated CCs.

### Cumulus cell-related genes are differentially expressed in NSN-CCs compared to SN-CCs

Among the differentially expressed genes brought out by our microarray analysis, we focused on two, *Amh* and *Has2*, both included as part of the ‘Reproduction’ biological process (Table 1) and known players of the CCs biology (Additional file 5). The *Amh* (Anti mullerian hormone) gene, whose protein inhibits the stimulatory effect of FSH (Follicle Stimulating Hormone) on follicle growth [16], was the most up-regulated in our experiments with a 2.34-fold higher expression in CCs of developmentally incompetent (NSN-CCs) compared to that of developmentally competent (SN-CCs) oocytes. The *Has2* (Hyaluronan synthase 2) gene, a CC-related transcript implicated in the secretion of hyaluronic acid, was 1.38-fold down-regulated in NSN-CCs *vs.* SN-CCs; its expression level, together with that of other CC-related transcripts, is directly correlated to bad preimplantation development and pregnancy outcome in human [17-23] and in cow [24,25].

Following, to confirm our microarrays results, we analysed by *q*RT-PCR the profile of expression of *Amh* and *Has2*; also, we extended this analysis to three more CC-related genes (not included in the microarrays list of differentially expressed genes likely because of the very low detection value scored, see data deposited in GEO, accession number GSE46906) previously shown to play a critical role during the latest phases of oocyte maturation and whose expression pattern was also correlated to the developmental outcome: *Ptx3* (Pentraxin 3), *Tnfaip6* (Tumor necrosis factor alpha-induced protein 6) and *Ptgs2* (Prostaglandin-endoperoxide synthase 2), associated with the stabilisation of the matrix at the time of cumulus expansion [17-19,21,25,26].

**Figure 2:**
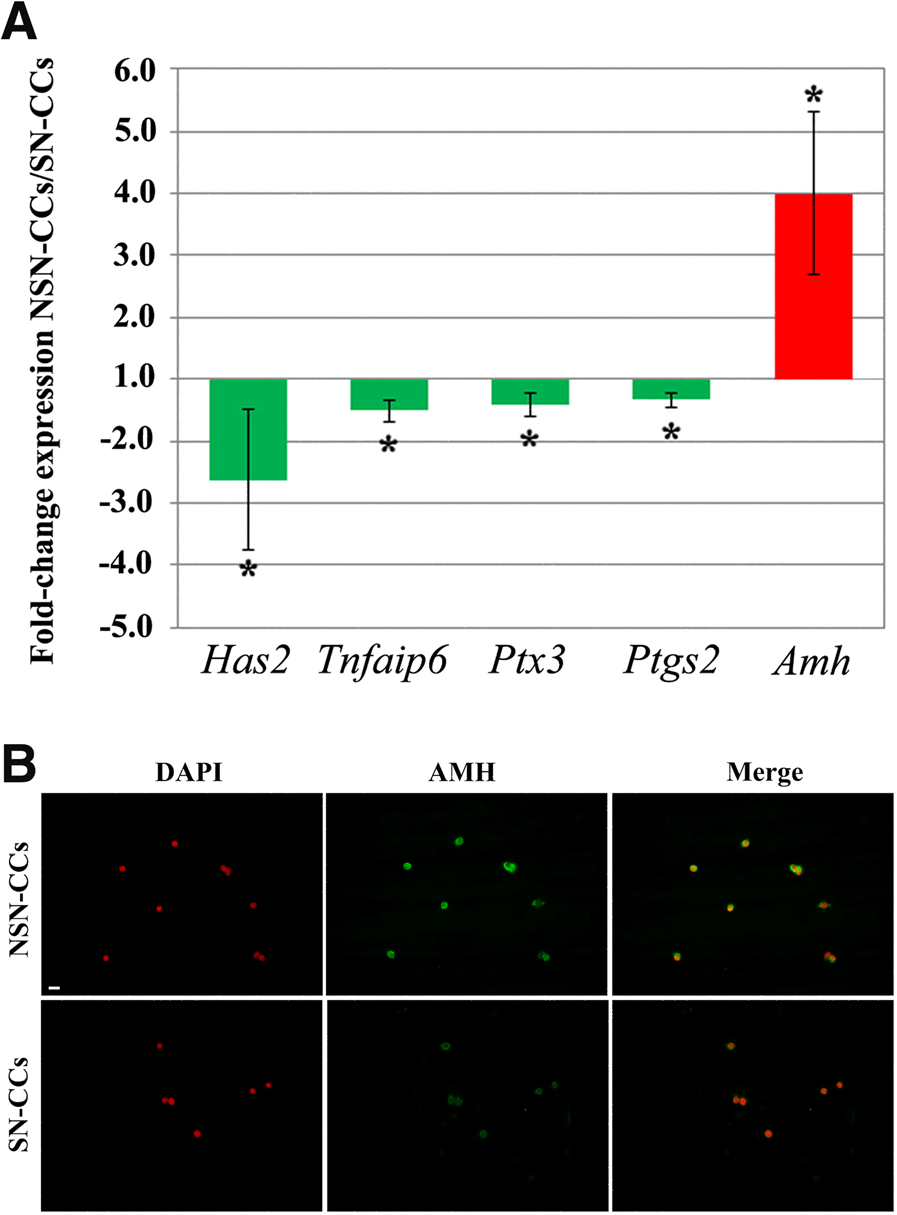
**Profile of expression of CC-related genes and immunofluorescence detection of AMH protein in NSN-CCs and SN-CCs.** (**A**) *q*RT-PCR analysis of the profile of expression of *Has2, Tnfaip6*, *Ptx3*, *Ptgs2* and *Amh* genes in NSN-CCs and SN-CCs. Fold-change expression in the comparison between NSN-CCs *vs.* SN-CCs. *, significantly different (*p*-values are reported in the text). (**B**) Immunofluorescence detection of the AMH protein in NSN-CCs and SN-CCs. Bar, 5 μm.

When comparing NSN-CCs *vs.* SN-CCs, *Has2* resulted the most down-regulated (-2.62-fold; *p* = 0.029), confirming the microarray results; followed by *Tnfaip6* (-1.51-fold; *p* = 0.008); *Ptx3* (-1.41-fold; *p* < 0.001) and *Ptgs2* (-1.34-fold; *p* = 0.007) (Figure 2). These results show that the profile of expression of mouse *Has2*, *Tnfaip6*, *Ptx3* and *Ptgs2* resembles that described earlier in human and cow CCs, i.e., lower in developmentally incompetent *vs*. competent oocytes [17-19,21,25,26]. The down-regulation of all these transcripts in NSN-CCs correlates with the poor quality of the enclosed oocyte [4,14] and is indicative of an overall immaturity/incompetence of these antral follicles. The *q*RT-PCR of the *Amh* transcript substantiated the microarrays data

**Table 1:** List of 247 differentially expressed genes within each of the 11 main biological processes

**Gene symbol**	**Biological process**	**Number of genes**
*Agrp, Ahrr, Arhgap15, Bcar3, Cabp4, Ccbp2, Cenpj, Clec4a3, Cx3cr1, Dclk2, Dnaja1, Edg2, Emr1, Enpp2, F10, Ffar3, Fgf15, Fgf5, Freq, Gabrb2, Gem, Gnat3, Gpr182, Gpr85, Grb7, Htr4, Ifnz, Irs1, Kremen2, Lag3, Ms4a10, Npffr2, Olfr104, Olfr1040, Olfr1105, Olfr1208, Olfr1219, Olfr1347, Olfr1383, Olfr1424, Olfr244, Olfr323, Olfr725, Olfr915, Omp, Oscar, Pdyn, Plcg2, Ppp1r1a, Ppp1r1b, Pth2, Ptpn6, Rab9b, Rassf9, Rgma, Rgs6, Rgs9, Rgs9bp, Rin1, Rl1, Rsu1, Sag, Shc4, Tmod2, Trem1, Treml2, Trpc5, Trpm3, V1rh5, Vmn2r42, Zap70, Zcrhav1*	Signal transduction	72
*Batf2, Chd1l, Cux2, Dhfr, Dis3, Ell3, Fhit,****Giyd2,****Gtf2h5, Gtf3c6, Hmbox1,****Hspa1a,****Il33, Kif18a, Larp6, Mef2b, Nono, Orc4l, P42pop, Rbm35a, Sp5, Tasp1, Tuba4a, Utp15, Zfp1, Zfp526, Zfp786, Zscan2*	Nucleic acid metabolism	28
*493444A2Rik, Agbl1, Alg11, B3gnt8, Cct6b, Cpeb2, Ctrl, Dnajc4, Dph5, EG623661, Eif3h, Eif4e, Fbxo44, Hipk4, Klhl13, Mrpl15, Mrpl39, Otub2, Rpl22l1, Senp6, Sh3d19, St3gal1, St8sia4, Tor2a, Usp45, Wnk2*	Protein metabolism	26
*Aif1, Alox12, Bik, Bnipl, C5ar1, Cacybp, Casp1, Casp4, Dapk2, Grid2, Isg20l1,****Lgmn,****Moap1, Nuak2, Pdcl3, Srgn, Tnfrsf4, Tnfsf13b, Tns4, Tpt1, Ube2z, Ywhae*	Cell death	22
*A930038C07Rik, Azgp1, Cdh1, Cdh11, Cdh13, Cdh3, Cldn3, Cml4, Col12a1, Col3a1, Ctnna3, Lypd3, Magi3*, *Mia1, Myh8*, *Pcdhb20, Pcdhb4, Serpini1, Spon2, Tecta, Utrn, Zan*	Cell adhesion	22
***Amh, Cited2,****Dazl, Fgf17, Fkbp6, Ggnbp1, Ghrh, Gnrhr, Has2, Hsf2, Mtl5, Pgr, Plau, Sox8, Spag6, Tcp11, Theg, Vmo1*	Reproduction	17
*AI428936, Abca5, Dixdc1, Dlk2,****Erdr1,****Fig4, Gdf10, Irf8, Lhx3, Lrg1, Neu2, Nkx2-3, Plk4, Rpgr,****Vim***	Cell differentiation	15
*Abcc2, Atp5e, Cox7b, Fxyd1, Gjb1, Hvcn1, Kcns1, Kctd7, Ranbp2, Slc25a13, Slc29a4, Slc39a1, Timm22*	Transmembrane transport	13
*Actl6b, Amn, Bin3, Cdx1, Cftr, Des, Ebf1, Eln, Fnbp1, Hydin, Myl6b, Prr15*	Development	12
*Alox5ap, Cpt1c, Cyp4f14, Elovl2, Fabp5, Idi1, Ihpk3, Ndufab1, Pigw, Pnliprp2, Ptgds*	Lipid metabolism	11
*Cdkn2a, Gbx2, Gm606, Il13, Pttg1, Tnfrsf9, Txnrd1, Wfdc1*	Cell cycle	8

Regular font, down-regulated genes; bold font, up-regulated genes.

 showing an even higher average (4-fold) up-regulation in NSN-CCs compared to SN-CCs (*p* < 0.05) (Figure 2A). This differential expression was further confirmed at the protein level. Imunofluorescence detection of the AMH protein revealed that NSN-CCs display very bright fluorescence, whereas SN-CCs show a faint signal (Figure 2B).

## Conclusions

In this study, the classification of mouse antral oocytes, based on their chromatin organisation into developmentally competent (SN) and incompetent (NSN), allowed the separation of their surrounding CCs into two distinct groups. For the first time, the microarray-based evaluation of their transcriptome highlighted a similar transcriptional activity, with the exception of 422 genes, 97.6% of which are down-regulated in NSN oocytes. The differential gene expression between the two types of CCs includes a group of four down-regulated CC-related (*Has2, Ptx3, Tnfaip6* and *Ptgs2)* and one up-regulated (*Amh*) gene in NSN-CCs compared to SN-CCs. When analysed by *q*RT-PCR, *Amh* is the most differentially expressed with an average 4-fold higher expression in CCs associated with NSN oocytes, demonstrating its robustness as a further non-invasive diagnostic marker for the selection of developmentally competent or incompetent mouse antral oocytes. This result assumes a noteworthy significance at the light of a very recent study showing that human *AMH* is highly expressed in CCs of preovulatory follicles containing premature or atretic oocytes [27]. It would be extremely interesting, for the aim we are pursuing of identifying non-invasive markers of developmental competence, to correlate the expression of this gene with the chromatin configuration in these human oocytes.

Overall, the regulated nature of the identified group of genes may represent the molecular signature of CCs associated either with developmentally incompetent or competent oocytes. Hence, our data might represent a valuable resource for enabling the development of novel non-invasive molecular tools for the assessment of oocyte quality and for the detailed investigation of the complex bi-directional interactions occurring between CCs and oocytes.

## Methods

### Animals and hormonal treatment

Four to six week-old B6C3F1 female mice (Charles River, Como, Italy) were injected intraperitoneally with 3.75 IU folligon (Intervet, Italy) 48 hours before sacrifice. Animals were maintained in the department animal facility under controlled conditions: 22°C of temperature and a 12/12 hr dark/light cycle. This study was carried out in strict accordance with the protocol approved by our University and the European (n. 86/609/CEE) and Italian (n. 116/92, 8/94) legislation. The protocol was approved by the Committee on the Ethics of Animal Experiments of the University of Pavia (Protocol Number: 1–2010).

### Isolation and manipulation of antral cumulus-oocyte-complexes

Cumulus-oocyte-complexes (COCs) were released in M2 medium [28] at 37°C by puncturing the ovarian surface with a sterile 26G needle. Using a mouth-controlled sterile Pasteur micropipette each COC was transferred into a 7 μl drop of fresh M2 medium and their enclosed oocytes were separated from the CCs by gently pipetting in and out the COC. Single oocytes were then classified as NSN or SN depending on their chromatin organization, as described before [4,14]. Briefly, each oocyte was transferred into a 5 μl droplet of M2 medium containing the fluorochrome Hoechst 33342 (50 ng/ml) and incubated at 37°C for 10 min. After staining, oocytes were classified as belonging to the SN or the NSN type, according to the presence or absence, respectively, of a ring of Hoechst-positive chromatin surrounding the nucleolus. Classification was performed with an Olympus Provis fluorescence microscope. Based on this classification, the CCs belonging to NSN (NSN-CCs) or SN (SN-CCs) oocytes were separately collected. For microarrays analysis a total of 638 NSN and 1769 SN COCs were isolated from 234 ovaries during 21 separate experiments. NSN-CCs or SN-CCs were pooled together into two batches each, representing biological replicates. *q*RT-PCR analysis was performed on RNA extracted from NSN-CCs or SN-CCs isolated from ~ 40 COCs. NSN-CCs and SN-CCs were transferred into distinct 1.5 ml Eppendorf tubes containing each 500 μl 1× PBS, centrifuged at 13.000 rpm for 3 min and the pellets flash-frozen in liquid nitrogen and stored at -80°C.

### RNA extraction

RNA was extracted using the RNeasy Mini Kit (Qiagen, USA) following the manufacturer’s instructions. To eliminate genomic DNA contamination, on-column DNase digestion (Qiagen) was performed. The isolated total mRNA was quality checked by Nanodrop analysis (Nanodrop Technologies, Wilmington, DE, USA).

### Microarray analysis

RNA for microarray analysis was obtained following a two-round linear amplification with Illumina TotalPrep RNA Amplification Kit (Ambion, Austin, TX, United States) performed with 400 ng mRNA, which is a complete system for generating biotin-labelled cRNA for hybridization with Illumina Sentrix arrays. For each sample, 1.5 μg cRNA was hybridized onto Illumina mouse-8 BeadChip version 3. After hybridization, washing and Cy3-streptavidin staining, the scanning was performed. All these passages have been performed on the Illumina Bead-Station 500 platform (Illumina, San Diego, CA, United States) with reagents and following the protocols supplied by the manufacturer. Two biological replicates of SN-CCs and NSN-CCs were hybridised. All basic expression data analysis was carried out using the manufacturer’s software BeadStudio 1.0. Raw data were background-subtracted and normalised using the “rank invariant” algorithm. Normalized data were then filtered for significant expression on the basis of negative control beads. Selection for differentially expressed genes was performed using arbitrary thresholds for fold changes and statistical significance, computed through a permutation-based test. The GO enrichment analysis was performed using the tools provided by the data mining and bioinformatics software Orange (http://orange.biolab.si/). Pathways and networks were obtained using Orange and David (http://david.abcc.ncifcrf.gov/). A MeSH annotation analysis was performed through a literature-based search strategy as previously described [29] (for details see Additional file 6). The data have been deposited in GEO (accession number GSE46906).

### Reverse-transcription and real time PCR

The RNA extracted from pooled NSN-CCs or SN-CCs, was reverse transcribed into cDNA using the following mix of reagents (Applied Biosystems, Italy) in a 20 μl reaction mixture: 3 μl of RNA, 1× PCR buffer, 1 mM MgCl^2^, 2 mM of each dNTP, 2.5 μM oligo d(T)_16_, 20 U RNase Inhibitor, 50 U MuLV reverse transcriptase. The reverse transcription was performed at 25°C for 10 min, 42°C for 15 min, 99°C for 5 min, on a GeneAmp 9700 thermocycler (Applied Biosystems). One twentieth of the resulting cDNA product was amplified in duplicate by *q*RT-PCR in 20 μl reaction mixture with 200 nM of each specific primer designed using the program Primer3 (Additional file 7) and the MESA GREEN qPCR MasterMix Plus for SYBR assay no ROX sample (Eurogentec, Italy) at 1× final concentration. The amplification reaction was performed in a Rotorgene 6000 (Qiagen, USA) as follows: 95°C for 5 min, followed by 40 cycles at 95°C for 10 sec, 60°C for 15 sec, 72°C for 20 sec. The Rotorgene 6000 Series Software 1.7 was used for the comparative concentration analysis. Prior to the *q*RT-PCR analysis of the five genes under study, we searched for a reference gene that was equally expressed in samples of 100 NSN-CCs or SN-CCs. Among several gene sequences tested, *β-actin* (*Actb*) gave an equivalent profile of expression in the two types of CCs and thus was used for the samples normalisation. *q*RT-PCR amplification was performed on cDNA from three independent experiments and the Student’s *t*-test employed to analyse the results. Differences were considered significant when *p* < 0.05 and data are expressed as frequency mean ± standard deviation (S.D.).

### Immunocytochemistry

After isolation, NSN-CCs or SN-CCs were collected in 1.5 ml Eppendorf tubes and 80 μl fresh M2 medium was added. Cells were cytospun onto a glass slide at 1000 rpm for 10 min, followed by 2000 rpm for 10 min and 5000 rpm for 15 min. Then, they were washed in PBS, prior to fixation with 4% paraformaldehyde in PBS for 20 min. Then, cells were washed with PBS and incubated with primary goat polyclonal AMH antibody (C-20) (Santa Cruz Biotechnology; SC-6886; 1:100 in PBS) at 4°C overnight. After three washes in 0.1% Tween PBS for 10 min, cells were incubated with the secondary antibody Alexa 555-conjugated donkey anti-goat antibody (1:500 in 0.1% Tween PBS; Invitrogen, Italy) at 37°C for 1 hr. At the end of incubation, cells were washed three times for 10 min each with 0.1% Tween PBS and nuclei counterstained with DAPI (0.2 μg/ml in PBS) for 10 min. Finally, glass slides were mounted in Vectashield (Vector, United Kingdom) and examined with an Olympus BX60 epifluorescence microscope equipped with single-bandpass filters for DAPI and Alexa555. Negative controls were treated with the same procedure, except for the incubation with the primary antibody. Digital images were obtained with an Olympus DP72 digital camera and processed using Adobe Photoshop software.

## Competing interests

The authors declare that they have no competing interests.

## Authors’ contributions

GV and VM did the isolation of all the COCs and CCs, immunocytochemistry and RT-PCR analyses; AP carried out the microarray analyses; FM, LS, MG and RB did the bioinformatics analyses; CAR and GM worked on the elaboration of the study; JA conceived the study, participated in its design and coordination, did the microarrays analyses and worked on drafting the manuscript, SG and MZ conceived the study, participated in its design and coordination, participated in the bioinformatic analyses and worked on drafting the manuscript. All authors read and approved the final manuscript.

## Supplementary Material

Additional file 1 **List of regulated genes.** List of down- (green) and up- (red) regulated genes in the comparison between NSN-CCs *vs*. SN-CCs. Click here for file

Additional file 2 **Annotation-based gene networks.** Node colours indicate the main GO biological process related to each gene, the size is adjusted proportionally to the Betweenness Centrality and increasing line width indicates stronger annotation relationship. (A) *Pttg1* gene network made of 142 genes. (B) *Pttg1* gene network made with its two more proximal neighbours. Click here for file

Additional file 3 **Interrogation of MeSH and STRING repositories.** Genes belonging to the network originated from the analysis of the group of 247 regulated genes. Genes in green, down-regulated; genes in red, up-regulated. Click here for file

Additional file 4 **Lists of keywords related to folliculogenesis.** MESH_B: set of basic key annotations. MESH_BC: initially selected key terms plus their child terms. MESH_BCP: initially selected key terms plus their parent terms. Click here for file

Additional file 5 **Bibliographic analysis.** List of genes and their corresponding scores and p-values obtained for each set of key terms (MESH_B, MESH_BC, MESH_BCP) by means of the MeSH annotation analysis (see M&M). Results include the number of folliculogenesis key terms retrieved in the complete list of gene annotations (MESH), the number of key terms included in the most relevant terms (TFIDF), the value of the score that reflects the association of the gene to folliculogenesis keywords and its associated *p*-value (PVALUE_TFIDF). Numbers of terms rated as relevant by TFIDF (NUM_TFIDF) and retrieved articles (NUM_ARTICLES) are also indicated. In yellow, genes showing a *p*-value < 0.05 in all the three sets of key terms. Click here for file

Additional file 6 **Methods.** MeSH annotation analysis. Click here for file

Additional file 7 **Primer sequences.** List of primer sequences used for the *q*RT-PCR. F, forward primer; R, reverse primer. Click here for file
