# Supplementary figures and images for: Transcriptome based identification of mouse cumulus cell markers that predict the developmental competence of their enclosed antral oocytes
Source: BMC Genomics. 2013 Jun 7;14:380. doi: 10.1186/1471-2164-14-380 (PMC3679864; doi:10.1186/1471-2164-14-380)

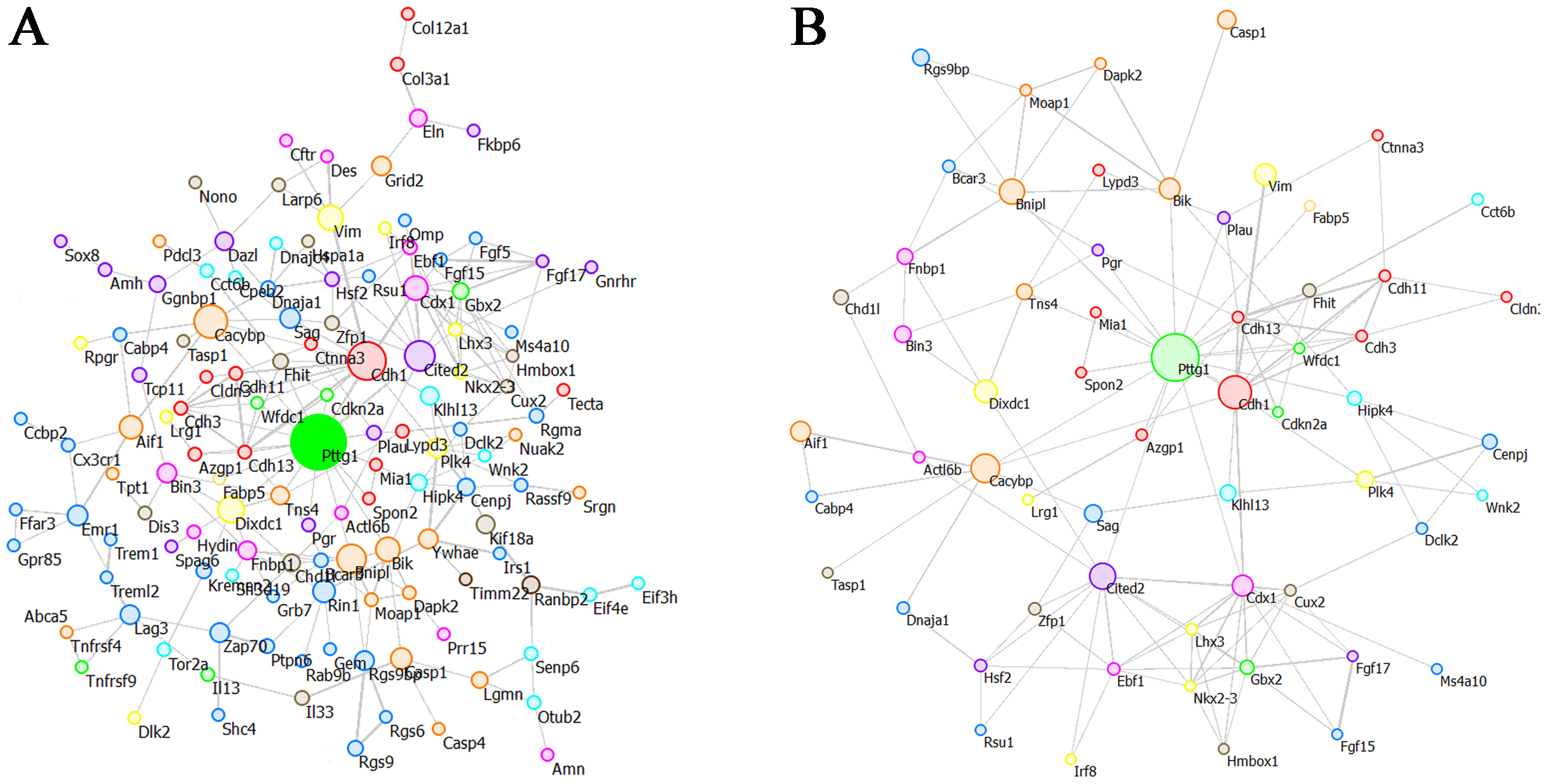

Supplement: Additional file 2 — Annotation-based gene networks. Node colours indicate the main GO biological process related to each gene, the size is adjusted proportionally to the Betweenness Centrality and increasing line width indicates stronger annotation relationship. (A) Pttg1 gene network made of 142 genes. (B) Pttg1 gene network made with its two more proximal neighbours. [file 1471-2164-14-380-S2.jpeg]
