# Supplementary material for: Transcriptome based identification of mouse cumulus cell markers that predict the developmental competence of their enclosed antral oocytes
Source: BMC Genomics. 2013 Jun 7;14:380. doi: 10.1186/1471-2164-14-380 (PMC3679864; doi:10.1186/1471-2164-14-380)
Supplement: Additional file 6 — Methods. MeSH annotation analysis. [file 1471-2164-14-380-S6.doc]

**Methods**

***Mesh annotation analysis***

We assigned a list of MeSH annotation terms to each differentially regulated gene. The search methodology exploits the NCBI Web Services to retrieve the most recent literature about specific genes from PubMed. The annotations assigned to each regulated gene included all the MeSH terms used for indexing the related articles. A text mining technique, known as TF-IDF (Nuzzo *et al.,* 2010), was exploited to rank the lists of terms according to their relevance, obtaining a subset of MeSH terms specifically associated to each gene. Then, the information extracted from the literature was combined with expert knowledge on the domain. Starting from a list of keywords related to folliculogenesis (Table 1S), we extracted a set of basic key annotations (*MESH_B*) by identifying the corresponding terms in the MeSH database. An additional list of key terms (*MESH*_*BC*) was obtained by including the initially selected key terms’ subordinate in the MeSH hierarchy, each subordinate being a more specialized term commonly referred to as child term. Finally, we added the parent terms of all the annotations in MeSH_B, i.e. their directly connected superior terms in the hierarchy, obtaining a third subset of key annotations (*MESH*_*BCP*). In order to account for the involvement of each regulated gene in the domain of interest, we developed a score based on the number of its associated key terms. Three different scores were computed, one for each of the considered set of key annotations. For each gene, we computed a first sub-score as the proportion of key terms included in the complete list of MeSH terms associated with that gene. A second sub-score was obtained by evaluating the proportion of key terms included in the 10% top-relevant terms, ranked according to TF-IDF. To compute the final score, we combined the two sub-scores by applying a weighted sum of the two contributions, where a higher weight was assigned to the second sub-score. This procedure allowed focusing on the presence of key terms in the subset of top relevant terms, rather than simply considering keywords retrieved in the entire list of annotations. For each gene and each score, the probability of obtaining a score at least as high as the one observed was assessed by means of a permutation strategy. Genes with p-values ≤ 0.05 for all the three scores were considered significantly associated to the folliculogenesis keywords.

The MeSH annotation terms were combined with information on protein associations provided by the STRING database (http://string-db.org) to generate gene networks. For every pair of regulated genes, a connecting link in the network was added if the MeSH annotation similarity or the confidence score assigned by STRING to the association exceeded a cut-off value of 0.6. To quantitatively characterize the importance of the nodes in the networks, we computed a topological index known as Betweenness Centrality (Yu *et al.,* 2007). This measure allows identifying genes that make possible the communication among clusters of nodes in the network characterized by high internal connectivity.
