# Supplementary material for: Transcriptome based identification of mouse cumulus cell markers that predict the developmental competence of their enclosed antral oocytes
Source: BMC Genomics. 2013 Jun 7;14:380. doi: 10.1186/1471-2164-14-380 (PMC3679864; doi:10.1186/1471-2164-14-380)
Supplement: Additional file 7 — Primer sequences. List of primer sequences used for the qRT-PCR. F, forward primer; R, reverse primer. [file 1471-2164-14-380-S7.doc]

**Additional file 7.** List of primer sequences used for the qRT-PCR. F, forward primer; R, reverse primer.

| **Primer** | **Sequence** | **Amplicon length (bp)** |
| --- | --- | --- |
| ***Has2* F** | 5’-AAGACCCTATGGTTGGAGGTG-3’ | 95 |
| ***Has2* R** | 5’-CATCCAGTATCTCACGCTGCT-3’ |
| ***Ptx3* F** | 5’-TGGACAACGAAATAGACAATGG-3’ | 95 |
| ***Ptx3* R** | 5’-GATGAACAGCTTGTCCCACTC-3’ |
| ***Ptgs2* F** | 5’-GAGTGGGGTGATGAGCAACTA-3’ | 96 |
| ***Ptgs2* R** | 5’-GCTCAGGTGTTGCACGTAGTC-3’ |
| ***Tnfaip6* F** | 5’-CGGGTATCATCGATTATGGAA-3’ | 100 |
| ***Tnfaip6* R** | 5’-TCTGTGAAGACACCACCACAC-3’ |
| ***Amh* F** | 5’-AGCAGGCCCTGTTAGTGCTAT-3’ | 94 |
| ***Amh* R** | 5’-CGAGTAGGGCAGAGGTTCTGT-3’ |
| ***Actb* F** | 5’-CGCGAGCACAGCTTCTTTGC-3’ | 90 |
| ***Actb* R** | 5’-GACGACCAGGCGAGCGATAT-3’ |
